# Resolution of large pelvic lymphocele after incidental intracystic hemorrhage caused by percutaneous catheter drainage: Case report

**DOI:** 10.1111/jog.15178

**Published:** 2022-02-02

**Authors:** Ai Nio, Masao Okadome, Kumi Shimamoto, Kenzo Sonoda, Toshiaki Saito

**Affiliations:** ^1^ Gynecology Service National Hospital Organization Kyushu Cancer Center Fukuoka City Fukuoka Japan

**Keywords:** autologous blood, blood patch, drainage, lymphocele/lymphocyst, sclerotherapy

## Abstract

We report the case of a large pelvic lymphocele after an ovarian cancer operation, which incidentally vanished after bleeding resulting from percutaneous catheter drainage. The patient was a 74‐year‐old woman with stage IVB ovarian cancer who underwent surgery including pelvic lymph node dissection. Three months after surgery, computed tomography revealed a large (13‐cm diameter) pelvic lymphocele with associated bilateral hydronephrosis and left femoral vein thrombosis. The lymphocele was repeatedly drained by percutaneous aspiration, and the day after the second procedure, the drainage fluid became bloody. The catheter was clamped for 3 days and then removed. The lymphocele volume gradually decreased, and it was not seen on a computed tomography scan 70 days after drainage. The lymphocele did not recur prior to her death. In this case, the intracystic hemorrhage was considered to have served as a blood patch for lymph leakage.

## Introduction

Pelvic lymphoceles are a common complication after lymphadenectomy for malignant gynecological tumors, and only a small fraction are symptomatic. However, some cases easily recur when treated by drainage alone; therefore, we consider another therapy. This is a rare case report of a large pelvic lymphocele after an ovarian cancer operation, which incidentally vanished after bleeding resulting from percutaneous catheter drainage.

## Case report

A 74‐year‐old woman was diagnosed with ovarian cancer stage mediastinal lymph node metastasis (cT3cN1M1), and she began chemotherapy with paclitaxel/carboplatin and bevacizumab. After four courses of treatment, she underwent total abdominal hysterectomy, bilateral salpingo‐oophorectomy, pelvic lymph node dissection, para‐aortic lymph node biopsy, and omentectomy. The left ureter was constricted by peritoneal dissemination. Multiple lesions smaller than 1 cm remained under the diaphragm and mesentery; as a consequence, the reduction rate was 60%.

**Figure 1:**
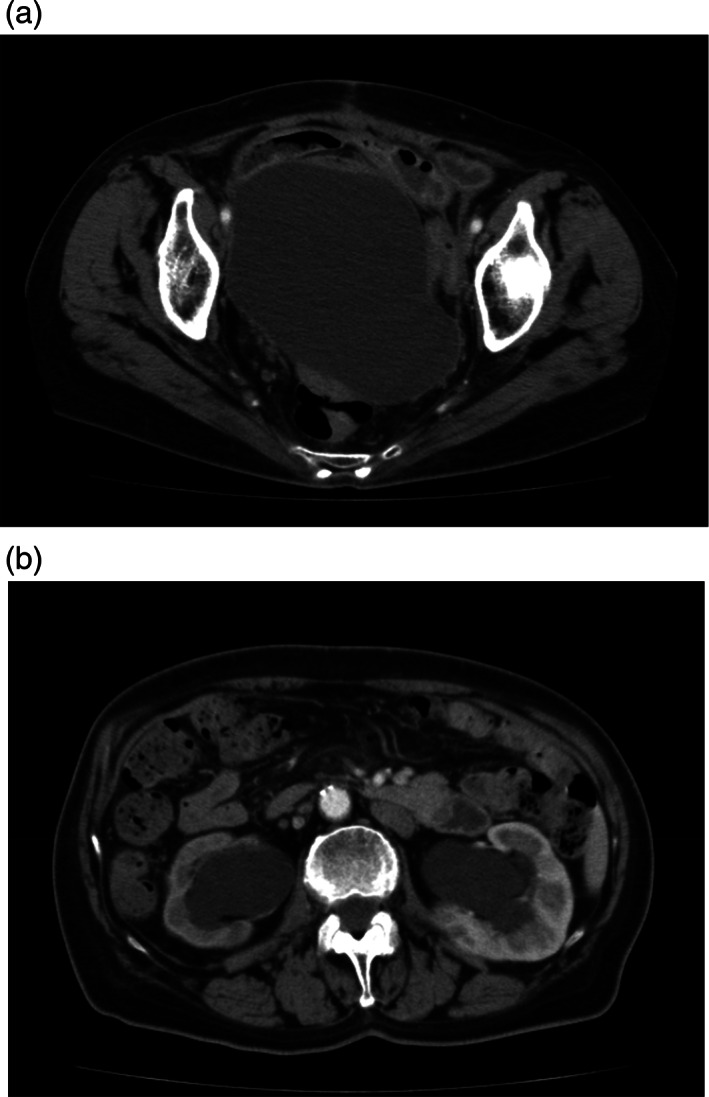
Three months after laparotomy including pelvic lymph node dissection, a large pelvic lymphocele (a) and bilateral hydronephrosis (b) were detected by computed tomography

Paclitaxel/carboplatin and bevacizumab were continued following surgery. Three months after the operation, she developed a large pelvic lymphocele with a diameter of 13 cm and bilateral hydronephrosis on computed tomography (CT) scan (Figure 1). Blood tests showed serum creatinine was 0.89 mg/dL and D‐dimer was 3.42 μg/mL. Leg vein ultrasound revealed thrombus in the left superficial thigh vein, and anticoagulant therapy using edoxaban was initiated. Seven months after the operation, the size of the pelvic lymphocele was 15 cm, and serum creatinine had risen to 1.35 mg/dL. She was symptomatic with abdominal distension and right thigh swelling. Percutaneous catheter drainage was performed with no complications, and the catheter (trocar aspiration kit 12 Fr, 4.2 mm; Covidien Japan, Inc., Minato City, Japan) was removed after 6 days without sclerotherapy. The amount of removed lymphatic fluid was 1235 mL (1050 mL on the first day), with negative cytological examination for neoplastic cells. The lymphocele temporarily shrank to 4 cm in diameter, in parallel with improvement in serum creatinine to 0.98 mg/dL, and her thigh swelling improved.

**Figure 2:**
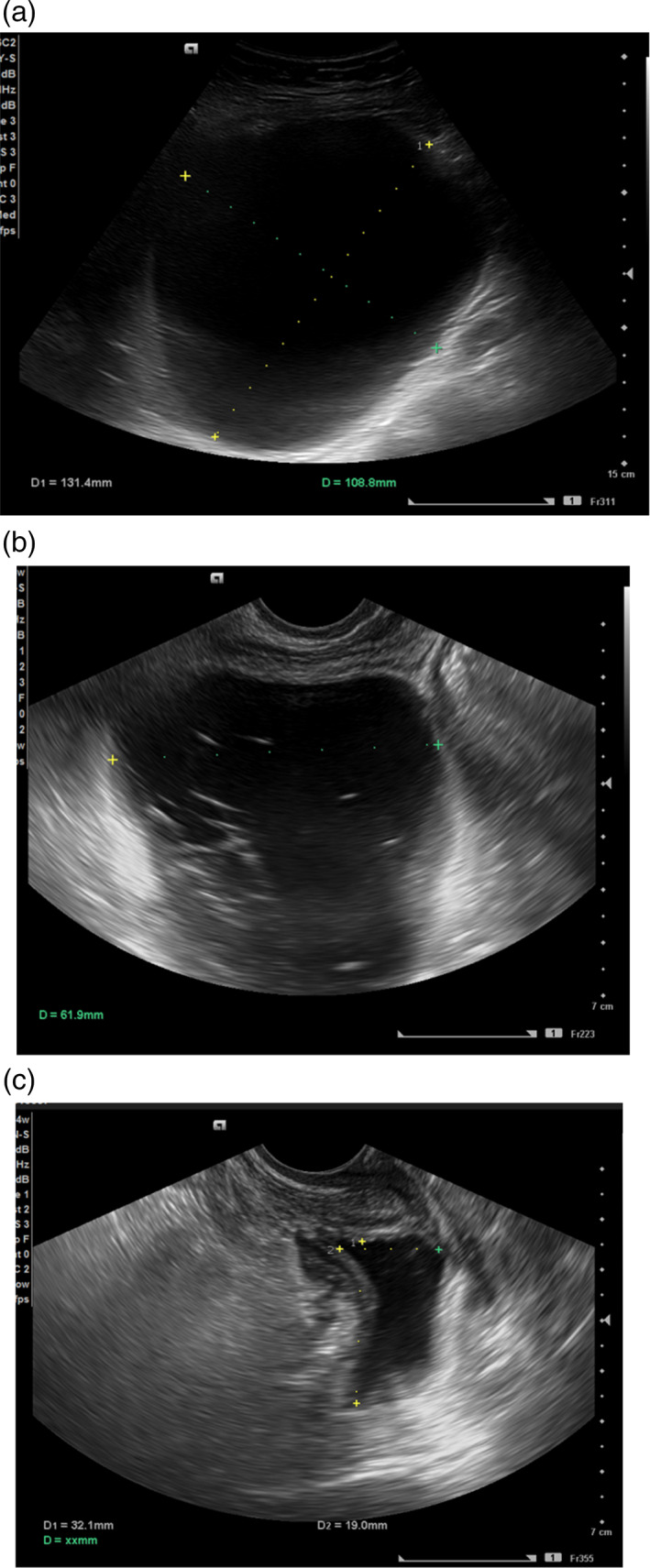
Ultrasound images of the recurrent lymphocele. Before drainage, the size was 13 × 11 cm. Its echogenicity by abdominal ultrasound was low (a). During drain clamping, the size was almost 7 cm. The echo image by transvaginal ultrasound showed a reticular pattern, indicating intracystic hemorrhage (b). One week after drain removal, transvaginal ultrasound revealed that the lymphocele had shrunk to 3 × 2 cm (c)

**Figure 3:**
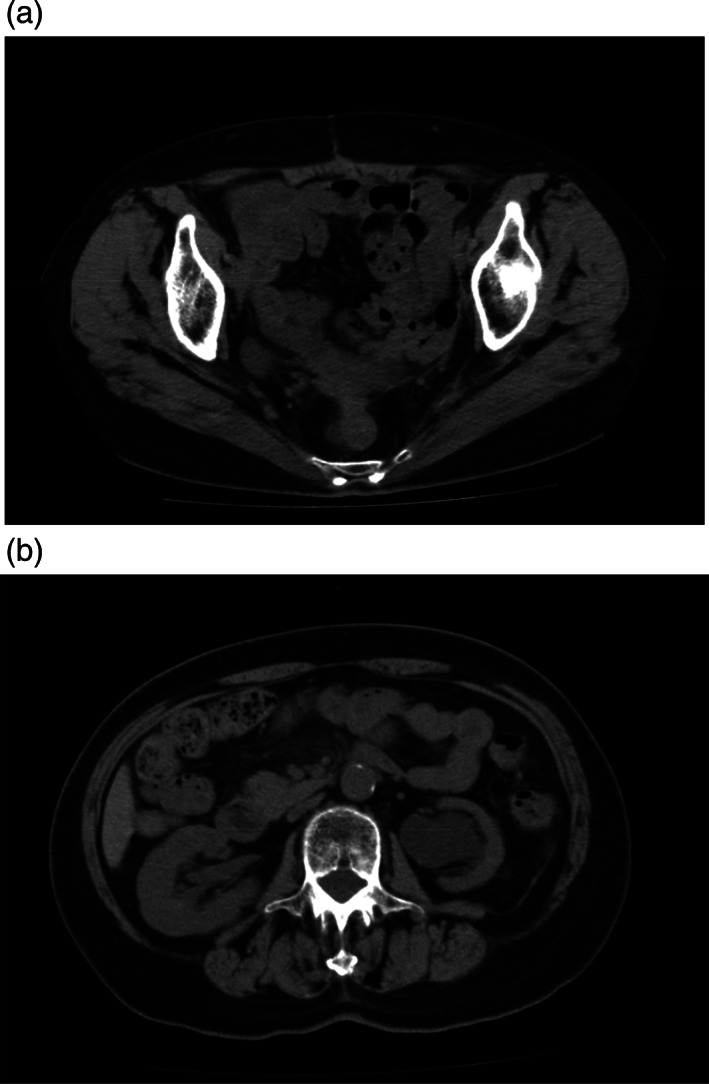
Four months after the second drainage, neither the lymphocele nor right hydronephrosis were detected by computed tomography (a, b)

However, the lymphocele regrew up to a diameter of 14.5 cm in 3 months, leading to the recurrence of abdominal distension and deterioration in renal function (serum creatinine 1.24 mg/dL). She underwent repeat percutaneous catheter drainage, without sonographic guidance after confirming the position by transabdominal ultrasound. Anticoagulant therapy was continued unchanged. The drained fluid was serous at first (870 mL), but it appeared lightly bloody in 1 h. A day and a half later, it appeared darkly bloody; the amount of fluid was 285 mL in 30 h. Anemia was diagnosed and was progressive (hemoglobin decreased from 7.0 to 5.3 g/dL). Her vital signs were stable, yet her estimated blood loss was 680 mL, calculated as 46.6 kg × 0.06 × (7–5.3)/7. We clamped the drain, stopped edoxaban the next day (15 h after clamping), and gave her a blood transfusion and tranexamic acid orally. We believed that the drain had hit the lymphocyst wall this time, and her renal dysfunction had intensified the effectiveness of edoxaban. The administration of bevacizumab may have affected her bleeding tendency. The drain was removed 3 days after clamping, with the expectation of achieving spontaneous hemostasis with the increased intracyst pressure. By ultrasound, the size of the lymphocele, including hemorrhage, was 90 × 54 mm initially; it decreased to 39 × 32 mm in 1 week (Figure 2). Accordingly, serum creatinine improved to 0.73 mg/dL. Both before and after this procedure, no significant systemic inflammation was observed. She had no fever, and bloods tests showed a white blood cell count of 4360–5770/μL and C‐reactive protein 0.39–1.19 mg/dL. After this procedure, she had no symptoms except for mild abdominal distension for a week. Four months after the second procedure, the lymphocele diminished and simultaneously the right hydronephrosis was improved on CT scan, whereas the left hydronephrosis persisted, similar to the preoperative state (Figure 3). Chemotherapy continued, but the treatment was ineffective, and she died 19 months after the second drainage (30 months after surgery). However, the lymphocele did not recur while she was alive.

Written informed consent was obtained from the patient for publication of this case report and accompanying images.

## Discussion

Lymphoceles are one of the most common complications of pelvic or para‐aortic lymphadenectomy for gynecological cancer. The reported frequency is 15%–20%, whereas symptomatic cases account for 4%–7%.^1^, ^2^, ^3^ Patients often present with abdominal distension, pain, edema, or constipation, as well as more severe symptoms such as infection, hydronephrosis, or deep venous thrombosis. As a conservative treatment, lymphoceles are primarily treated with percutaneous catheter drainage, and then sclerotherapy will be considered when they recur.

A large‐volume lymphocele requires a long‐term indwelling catheter, which confers an increased risk of infection and recurrence. When it is refractory to conservative treatment, we need to consider surgical therapies, such as embolization of the leaking lymphatic ducts, lymphatic microsurgery (lymphatic ligation, lymphovenous or lympholymphatic anastomosis).^3^, ^4^ Laparoscopic marsupialization is also an option.^5^ To identify and embolize lymph leakage, lymphangiography using indocyanine green or ethiodized oil is effective.^6^, ^7^ A systematic review^8^ showed proportions of successful interventions for percutaneous catheter drainage, percutaneous catheter drainage with the addition of sclerotherapy, and embolization: 0.612 (95% confidence interval [CI]: 0.490–0.722), 0.890 (95% CI: 0.781–0.948), and 0.922 (95% CI: 0.731–0.981), respectively. Sclerotherapy is more easily administered by gynecologists, compared with other surgical therapies.

Sclerotherapy induces an inflammatory reaction and specific apoptosis of the lymphatic endothelium. Povidone–iodine was used most often in the cohorts of the aforementioned systematic review. Pan et al.^9^ demonstrated the efficacy of percutaneous CT‐guided afferent lymphatic vessel sclerotherapy (using ethanol) after ineffective therapeutic transpedal lymphangiography. Semura et al.^10^ reported a retrospective comparative cohort study of sclerotherapy using OK‐432. The use of other sclerosing agents such as tetracycline, doxycycline, minocycline, and bleomycin has been reported.^3^ The adverse effects of sclerotherapy are pain, fever, erythema, flu‐like symptoms, and allergic reactions.^8^

Autologous peripheral blood has been used for the treatment of pneumothorax or malignant pleural effusion; the coagulated blood adheres to the peripheral tissue like an epidural blood patch in cerebrospinal fluid leakage, and symptoms are reduced. Only one report to date describes the usefulness of an autologous blood patch for the treatment of lymphocele. Nishibeppu et al.^11^ reported the case of a para‐aortic lymphocele treated with autologous peripheral blood injection. The patient presented with a small intestinal ileus because of compression by the lymphocele. The group chose treatment with autologous peripheral blood because it was less invasive in consideration of the nearby jejunum. After 100 mL of fluid was aspirated, a total of 35 mL of autologous peripheral blood was injected in four divided doses.

Similarly, in our patient, intracystic hemorrhage sealed the lymph leak, resulting in the disappearance of the large pelvic lymphocele in one procedure. It just happened to work fortuitously in our case, and so there was a non‐negligible limitation to this observation. However, the experience might support the effectiveness of autologous blood therapy for lymphocele treatment.

Our patient was treated with edoxaban. It has a short half‐life of about 10–14 h and needs about 24 h to clear sufficiently. It is just a speculation, but the blood in normal coagulation state that came out as bleeding in the cyst a day after stopping edoxaban might be effective for sealing the lymph leak, during clamping for 3 days. Nevertheless, it would have been supposed to be better to stop edoxaban before drainage of the lymphocele in terms of safety. Moreover, it is presumed that anticoagulant drugs should be discontinued especially in the case which autologous blood patch therapy is attempted.

Autologous peripheral blood is not a sclerosing agent, and so it is unknown how much blood and how long a period is needed for this treatment. It is expected that a certain degree of intracystic pressure is needed to seal the lymph leak, and thus it may require a moderate amount of blood to show effectiveness. However, as in the case of Nishibeppu et al.,^11^ autologous blood has the advantage of low irritation for peripheral organs. It may be an option for the treatment of symptomatic lymphocele, taking advantage of its lesser inflammatory response and greater cost effectiveness. However, further study is warranted to evaluate the effectiveness of autologous blood therapy for lymphocele.

## Conflict of interest

The authors declare no conflicts of interest associated with this manuscript.

## Author contributions

All authors read and approved the final manuscript.

## Data Availability

Data openly available in a public repository that issues datasets with DOIs
